# Molecular characterisation of *Galba truncatula*, *Lymnaea neotropica* and *L. schirazensis* from Cajamarca, Peru and their potential role in transmission of human and animal fascioliasis

**DOI:** 10.1186/1756-3305-5-174

**Published:** 2012-08-15

**Authors:** M Dolores Bargues, Patricio Artigas, Messaoud Khoubbane, Pedro Ortiz, Cesar Naquira, Santiago Mas-Coma

**Affiliations:** 1 Departamento de Parasitología, Facultad de Farmacia, Universidad de Valencia, Av. Vicente Andrés Estellés s/n, 46100 Burjassot - Valencia, Spain; 2 Facultad de Ciencias Veterinarias, Universidad Nacional de Cajamarca, Carretera Baños del Inca km 3,5, Cajamarca, Peru; 3 Instituto de Medicina Tropical "Daniel A. Carrión", Facultad de Medicina, Universidad Nacional Mayor de San Marcos, Calle José Santos Chocano 199, Lima 1, Perú

## Abstract

**Background:**

Human and animal fascioliasis is emerging in many world regions, among which Andean countries constitute the largest regional hot spot and Peru the country presenting more human endemic areas. A survey was undertaken on the lymnaeid snails inhabiting the hyperendemic area of Cajamarca, where human prevalences are the highest known among the areas presenting a "valley transmission pattern", to establish which species are present, genetically characterise their populations by comparison with other human endemic areas, and discuss which ones have transmission capacity and their potential implications with human and animal infection.

**Methods:**

Therefore, ribosomal DNA ITS-2 and ITS-1, and mitochondrial DNA 16S and *cox*1 were sequenced by the dideoxy chain-termination method.

**Results:**

Results indicate the presence of three, morphologically similar, small lymnaeid species belonging to the *Galba*/*Fossaria* group: *Galba truncatula*, *Lymnaea neotropica* and *L. schirazensis*. Only one combined haplotype for each species was found. The ITS-1, 16S and *cox*1 haplotypes of *G. truncatula* are new. No new haplotypes were found in the other two species. This scenario changes previous knowledge, in which only *L. viator* (= *L. viatrix*) was mentioned. *Galba truncatula* appears to be the most abundant, with high population densities and evident anthropophyly including usual presence in human neighbourhood. Infection by *Fasciola hepatica* larval stages were molecularly confirmed in two populations of this species. The nearness between *G. truncatula* populations presenting liver fluke infection and both human settings and schools for children, together with the absence of populations of other lymnaeid species in the locality, suggest a direct relationship with human infection.

**Conclusions:**

The geographical overlap of three lymnaeid species poses problems for epidemiological studies and control action. First, a problem in classifying lymnaeid specimens in both field and laboratory activities, given their transmission capacity differences: *G. truncatula* mainly involved in transmission to humans, *L neotropica* typically responsible for livestock infection, and *L. schirazensis* unable for transmission. Although several phenotypic characteristics may be helpful for a preliminary specimen classification, a definitive classification can only be obtained by marker sequencing. Aditionally, *L. schirazensis* increases the confusion, owing to its ability to mix with other *Galba*/*Fossaria* species and distort fascioliasis data such as transmission capacity and infection susceptibility. Second, a problem for epidemiological analysis, surveillance and control by methods as mathematical modelling and Remote Sensing - Geographical Information Systems. In Cajamarca, low resolution mapping may be insufficient, as already verified in Andean areas where different lymnaeid species overlap.

## Background

Fascioliasis is a parasitic disease transmitted by freshwater lymnaeid snails and caused by *Fasciola hepatica* distributed almost throughout and *F. gigantica* in large regions of Africa and Asia [1]. Distribution, both in space (latitudinal, longitudinal and altitudinal) and time (seasonal, yearly), of fascioliasis depends on the presence and population dynamics of the specific intermediate host or vector species in its turn linked to the presence of the appropriate water bodies and on adequate climate characteristics enabling fluke development [1,2].

Although livestock species play an important reservoir role [3], transmission studies have shown that the metacercarial infective stage from different origins, such as sheep, cattle, pig and donkey, represent similar infectivity sources [4,5]. On the contrary, the specificity of fasciolid species regarding given lymnaeid species [6] represent a crucial factor in establishing not only the geographical distribution of the disease in both animals and humans, but also prevalences and intensities due to more or less appropriate ecological characteristics (population dynamics, anthropophylic characteristics, type of water bodies, etc.) of the different lymnaeid intermediate host or vector species. That is why different lymnaeid species appear linked to the different transmission patterns and epidemiological scenarios of this very heterogeneous disease in humans [1,7]. Similarly as in other vector-borne diseases, this relationship supports the use of lymnaeids as biomarkers of the disease at both local and large scales and can thus be useful for the validation of mathematical modelling and remote sensing – geographical information system (RS-GIS) tools for the control of the disease [8,9].

In the Americas, the greatest problems are known in Andean countries. Peru appears as the country presenting a larger public health problem due to human infection by *F. hepatica*. Human fascioliasis has been diagnosed in inhabitants from almost all Andean areas, including from the Altiplano [10] up to inter-Andean valleys [11-13], and even urban areas surrounding the capital of Lima [14] and low altitude areas closer to the Pacific coast [15]. Many of these areas have proved to be human endemic. A rural population of almost 8 million people is estimated at risk in Peru [16]. Bolivia presents the endemic area of the Northern Altiplano with the highest human prevalences and intensities known [17-19]. In Chile, a human endemic area has been described and human cases are reported yearly [20,21]. Available data on human infection in Venezuela [22], Ecuador [23] and Argentina [24] also suggest that the respective real situations in these three countries may be underestimated.

The increasing importance of human fascioliasis does not only rely on the recent wide emergence it shows, but also on the results obtained in studies on pathogenicity [25-28] and immunity [29,30], according to which this disease appears to be pronouncedly more complicated and with a greater impact in long-term infection than what was believed until the 90s. The origin of the emergence of fascioliasis in recent years has been argued to be related to climate change, at least in part and in given countries [31,32], as a consequence of the high dependence of fascioliasis transmission and freshwater lymnaeid snails on climate and environmental characteristics [8,9,33].

Emergence, long-term pathogenicity and immunological interactions are in the background of the decision taken by the World Health Organization (WHO) to include this disease within the so-called neglected tropical diseases (NTDs). The great concern related to the epidemiological situations in many countries led WHO to launch a worldwide initiative against this disease [34,35]. The first step of this initiative was a pilot intervention in different countries selected according to their different epidemiological situations and transmission patterns [1,7].

Peru was one of the countries selected for priority intervention. Within the human fascioliasis high altitude transmission pattern related to *F. hepatica* transmitted by lymnaeid vectors of the *Galba*/*Fossaria* group, two different subpatterns have been distinguished in Peru [1,7]: a) the altiplanic pattern, with endemicity distributed throughout an area of homogeneous altitude and transmission throughout the whole year due to high evapotranspiration rates leading lymnaeid vectors to concentrate in permanent water bodies [2]; examples are the Northern Bolivian Altiplano and the Peruvian Altiplano of Puno; b) the valley pattern, with endemicity distributed throughout an area of heterogeneous altitude and seasonal transmission related to climate [36,37]; Peruvian examples are the valleys of Cajamarca and Mantaro.

The present article deals with the lymnaeid snail surveys performed in the human and animal endemic areas of Cajamarca. The aim of the present study is to analyse the DNA sequences obtained from lymnaeids collected, mainly in the neighbourhood of localities where human infection is known to be high. The purpose is to establish which lymnaeid snail species are present, perform a molecular characterisation of their populations in Cajamarca by comparison with other populations of the same lymnaeid species in other human endemic areas, and finally discuss which ones have disease transmission capacity and their potential implications with the disease in humans and animals. Therefore, four DNA markers were selected: the two internal transcribed spacers of the nuclear ribosomal DNA (rDNA ITS-2 and ITS-1), and two genes of the mitochondrial DNA such as the large subunit 16S codifying for rRNAs and the cytochrome c oxidase subunit I (mtDNA *cox*1) codifying gene [6,22,38-46]. DNA markers have also proved to be useful in Planorbidae, the other snail family of medical interest [47,48]. Similarly as in other invertebrates [49], in lymnaeids ITS-2 and ITS-1 appear to be the best markers at species and supraspecific levels, whereas 16S and *cox*1 are useful at infraspecific and population levels [42,46].

## Methods

### Lymnaeid snail materials

The snail specimens studied were collected in the field, from lymnaeid populations present in geographical areas of the Departamento de Cajamarca in the northern Andean part of Peru, with human infection and/or animal fascioliasis endemicity. The majority of the snail populations studied were found close to schools whose children proved to be infected [13] or around neighbouring villages.

For an estimation of lymnaeid population densities, the method of counting the number of snails in a marked area in a unit of time was used. Marked areas were mainly a segment of a canal or, less frequently, a portion of the edge of a pool. In each locality, snails were collected by the same four people during 1 hour, with the purpose of collecting as many specimens as possible, so as to increase the probability of detecting infected snails.

**Figure 1:**
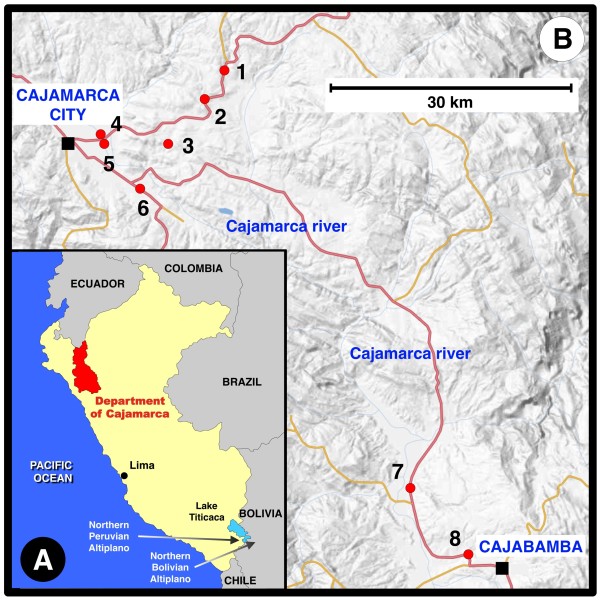
**Geographical distribution of lymnaeid sampling localities.** Maps showing location of the Department of Cajamarca within Peru (**A**) and fascioliasis endemic area of Cajamarca studied (**B**): 1 = Encañada, Encañada district; 2 = Santa Rosa de Chaquil, Encañada district; 3 = Tauripampa, Llacanora district; 4 = Baños del Inca (locality A), Baños del Inca district; 5 = Baños del Inca (locality B), Baños del Inca district; 6 = Yanamarca, Jesus district; 7 = Chaquicocha, Cajabamba district; 8 = Valle de Condebamba, Cajabamba district

Localities and their altitudes furnishing the lymnaeid specimens sequenced are noted in Table 1 and Figure 1. Snail specimens for molecular analyses, as well as trematode larval stages found in several of them, were fixed in 70% ethanol for DNA extraction procedures.

**Table 1:** Nuclear ribosomal and mitochondrial DNA haplotype code identification for lymnaeid species and populations studied from Cajamarca, Peru

**Lymnaeid species**	**Populations**				**rDNA ITS-2**		**rDNA ITS-1**		**mtDNA 16S**		**mtDNA *cox* 1**		**Combined H nomenclature**
	**Locality**	**Latitude (S)**	**Longitude (W)**	**Altitude**	**H**	**Acc. No.**	**H**	**Acc. No.**	H**	**Acc. No.**	H**	**Acc. No.**	
*G. truncatula*	Encañada, Encañada district N = 97/10/0; D = 9.7	07°05′21′	78°20′41″	3,130 m	1	AJ296271	E*	HE610430	B*	HE610432	d*	HE610435	G.tru-1E,16SB*,cox*1d
	Santa Rosa de Chaquil, Encañada districtN = 595/10/2; D = 119.0	07°07′48″	78°21′06″	3,038 m	1	AJ296271	E*	HE610430	B*	HE610432	d*	HE610435	G.tru-1E,16SB*,cox*1d
	Tauripampa, Llacanora district N = 452/10/2; D = 90.4	07°10′19″	78°24′26″	2,890 m	1	AJ296271	E*	HE610430	B*	HE610432	d*	HE610435	G.tru-1E,16SB*,cox*1d
	Baños del Inca (loc. B), Baños del Inca district N = 10/4/0; D = 10.0	07°10′14″	78°27′59″	2,655 m	1	AJ296271	E*	HE610430	B*	HE610432	d*	HE610435	G.tru-1E,16SB*,cox*1d
	Yanamarca, Jesus district N = 861/10/0; D = 287.0	07°12′54″	78°26′08″	2,633 m	1	AJ296271	E*	HE610430	B*	HE610432	d*	HE610435	G.tru-1E,16SB*,cox*1d
	Chaquicocha, Cajabamba district N = 15/4/0; D = 7.5	07°32′07″	78°08′58″	2,080 m	1	AJ296271	E*	HE610430	B*	HE610432	d*	HE610435	G.tru-1E,16SB*,cox*1d
*L. neotropica*	Valle de Condebamba, Cajabamba district N = 6/3/0; D = 6.0	07°36′16″	78°05′16″	2,390 m	1	AM412225	A	AM412228	A*	HE610433	a*	AM494008	L.neo-1A,16SA,*cox*1a
*L. schirazensis*	Baños del Inca (loc. A), Baños del Inca district N = 72/3/0; D = 14.4	07°09′44″	78°28′03″	2,665 m	1	JF272601	B	JF272604	A	JF272605	d	JF272610	L.schi-1B,16SA,cox1d

H = haplotype; N = lymnaeid specimens collected in each locality/ lymnaeid specimens sequenced/ lymnaeid specimens with *Fasciola hepatica* larval stages sequenced. . D = population density estimates for each locality, in number of lymnaeids collected by four persons per 1 m in 1 hour. * = new haplotypes for the corresponding lymnaeid species deposited in EMBL; ** = only preliminary haplotypes due to incomplete gene sequence. For the geographical situation of localities, see Figure 1.

### Molecular techniques

#### DNA extraction

The same procedure was performed for both lymnaeids and fluke larvae. The feet of lymnaeid snail specimens and trematode rediae found were suspended in 400 μl of lysis buffer (10 mM Tris–HCl, pH 8.0, 100 mM EDTA, 100 mM NaCl, 1% sodium dodecyl sulfate SDS) containing 500 μg/ml Proteinase K (Promega, Madison, WI, USA) and digested for 2 hr at 55°C with alternate shaking each 15 min. The procedure steps were performed according to methods outlined previously [6,50]. The extraction was then performed with phenol-chloroform and DNA was precipitated with ethanol. The pellet was dried and resuspended in 30 μl sterile TE buffer (pH 8.0). This suspension was stored at –20 °C until use.

#### DNA sequence amplification

Each one of the four DNA markers were PCR amplified independently for each lymnaeid specimen and each PCR product was sequenced for a bona-fide haplotype characterization. The complete sequences of the rDNA spacers ITS-2 and ITS-1 were amplified using primers previously described [6,41,50,51]. The complete ITS-1 of the trematode rediae was amplified by PCR according to methods outlined previously [51,52]. The target 16S gene region was amplified by PCR using a set of universal primers [53]. Amplification procedures and thermal cycler conditions were carried out as previously described for lymnaeids [38,42,44]. A mitochondrial DNA *cox*1 gene fragment was amplified using universal primers [54]. Amplifications were generated in a Mastercycle ep*gradient* (Eppendorf, Hamburg, Germany) using 4–6 μl of genomic DNA for each 50 μl PCR reaction. PCR conditions were 30 cycles of 30 sec at 94°C, 30 sec at 50°C and 1 min at 72°C, preceded by 30 sec at 94°C and followed by 7 min at 72°C for ITS-2 and ITS-1, and by 40 cycles of 30 sec at 90°C, 1 min at 48°C and 1 min at 72°C, preceded by 2.5 min at 94°C and followed by 10 min at 72°C for *cox*1. Ten μl of each PCR product was checked by staining with ethidium bromide on 1% Nusieve® GTG agarose (FMC) gel electrophoresis, using the Molecular Weight Marker VI (Boehringer Mannheim) at 0.1 μg DNA/μl as control.

#### Purification and quantification of PCR products

Primers and nucleotides were removed from PCR products by purification on Wizard™ PCR Preps DNA Purification System (Promega, Madison, WI, USA) according to the manufacturer's protocol and resuspended in 50 μl of 10 mM TE buffer (pH 7.6). The final DNA concentration was determined by measuring the absorbance at 260 and 280 nm.

#### DNA sequencing

The sequencing of the complete rDNA ITS-2 and ITS-1 and the fragments of the mtDNA 16S and *cox*1 genes was performed on both strands by the dideoxy chain-termination method [55]. It was carried out with the Taq dye-terminator chemistry kit for ABI 3730 DNA Analyzer (Applied Biosystems, Foster City, CA, USA), using PCR primers.

#### Sequence alignments

Sequences were aligned using CLUSTAL-W version 1.8 and MEGA 5.0, and assembly was made with the Staden Package [56]. Subsequently, minor corrections were manually introduced for a better fit of nucleotide correspondences in insertions/deletions (indels) and/or microsatellite sequence regions. Genetic distances were measured, using parameters provided by PAUP v.4.0b10.

#### DNA haplotype nomenclature

The codes for the sequences obtained follow the standard nomenclature proposed for lymnaeid snails previously [1,41,46]. It shall be noted that haplotype codes are only definitive in the case of complete sequences (ITS-2 and ITS-1 in the present study). When dealing with fragments or incomplete sequences, haplotype codes are provisional (16S and *cox*1 in the present study).

#### Lymnaeid sequence comparisons

The following sequences from GenBank-EMBL have been used for comparison analyses:

-rDNA ITS-2: *G. truncatula* H1 [EMBL: AJ296271], H2 [EMBL: AJ243017] and H3 (= *L. viatrix sensu* Ueno et al., 1975; = *L. cubensis sensu* Ueno et al., 1975) [EMBL: AJ272051] [6,50,51]; *L. cubensis* H1 [EMBL: AM412223], H2 [EMBL: FN182200], H3 [EMBL: FN182201] and H4 [GenBank: JF514088] [22,50]; *L. viator* H1 from the type locality Rio Negro, Argentina [EMBL: AM412224] [50] and H2 [GenBank: JN051366] [21]; *L. neotropica* H1 from the type locality of Lima, Peru [EMBL: AM412225] [50] and H2 [GenBank: JF514089] [22]; *L. schirazensis* H1 [GenBank: JF272601] and H2 [GenBank: JF272602] [42].

-rDNA ITS-1: *G. truncatula* HA [EMBL: AJ243018], HB [AJ296270], HC (= *L. viatrix sensu* Ueno *et al*., 1975; = *L. cubensis sensu* Ueno et al., 1975) [EMBL: AJ272052] and HD [GenBank: JF514090] [22,41,50,51]; *L. cubensis* HA from the type locality of Cuba [EMBL: AM412226], HB [EMBL: FN182202] and HC [EMBL: FN182203] [22,50]; *L. viator* HA from the type locality of Rio Negro, Argentina [EMBL: AM412227] [50] and HB [GenBank: JN051368] [21]; *L. neotropica* HA from the type locality of Lima, Peru [EMBL: AM412228] [50]; *L. schirazensis* HA [GenBank: JF272603] and HB [GenBank: JF272604] [42].

-mtDNA 16S rRNA gene: *G. truncatula* 16S-A from Spain [EMBL: HE610431] (new sequence obtained for comparison purposes); *L. cubensis* 16S-A from the type locality of Cuba [EMBL: FN182204] (new sequence obtained for comparison purposes); *L. viator* 16S-A from the type locality of Rio Negro, Argentina, and also Chile [EMBL: HE610434] (new sequence obtained for comparison purposes); *L. schirazensis* 16S-A [GenBank: JF272605] and 16S-B [GenBank: JF272606] [42]; and other proximal species of the *Galba*/*Fossaria* group such as *Fossaria bulimoides* [GenBank: AF485657] and *F. obrussa* [GenBank: AF485658] [57].

-mtDNA *cox*1 gene: *G. truncatula cox*1a [EMBL: AM494011) [50], *cox*1b [GenBank: JF461487] [22], cox1c [GenBank: JN051372] [21] and *G. truncatula* [GenBank: EU818799] [58]; *L. cubensis cox*1a from the type locality of Cuba [EMBL: AM494009] [50] and *cox*1b [GenBank: FN182205] [22]; *L. viator* cox1a from the type locality Rio Negro, Argentina [EMBL: AM494010] [50], cox1b [GenBank: JN051373] and cox1c [GenBank: JN051374] [21]; *L. neotropica cox*1a from the type locality of Lima, Peru [EMBL: AM494008], *cox*1b [EMBL: FN356741], *cox*1c [GenBank: JF461485] and *cox*1d [GenBank: JF461486] [22,50,52]; *L. schirazensis cox*1a [GenBank: JF272607], *cox*1b [GenBank: JF272608], *cox*1c [GenBank: JF272609] and *cox*1d [GenBank: JF272610] [42].

#### Trematode sequence comparisons

The rDNA ITS-1 sequence of the trematode larval stages was compared with the following sequences from GenBank-EMBL:

-rDNA ITS-1: *F. hepatica* from Spain, France, Poland, Ireland, Iran, Japan, Korea, Vietnam, Australia, Egypt, Bolivia, Peru, Uruguay, Argentina, Chile, Venezuela, Ecuador and Mexico [GenBank: AB207139; GenBank: AB207140; GenBank: AB207141; GenBank: AB207145; GenBank: AB211236; GenBank: AB385611; EMBL: AJ243016; GenBank: EF612468; GenBank: EF612469] [1,51].

## Results

### Lymnaeid populations and their densities

Populations of lymnaeid species found are noted in Table 1, including coordinates, altitudes, number of lymnaeid specimens collected and population densities. Within a high altitude range between 2,080 and 3,130 m, the very high population densities, in localities such as Santa Rosa de Chaquil, Tauripampa and Yanamarca, are worth mentioning.

### Molecular characterisation of lymnaeids

Nuclear rDNA ITS-2 and ITS-1 and mtDNA 16S and *cox*1 nucleotide sequence data reported in this study are available in the GenBank™, EMBL and DDBJ databases under the accession numbers noted in Table 1. Initial snail species classifications, based on previous complete morphological decriptions [42,50], were subsequently verified by sequence comparisons, as noted in the following.

#### Galba truncatula

Specimens from six different populations found in the localities of Encañada, Santa Rosa de Chaquil, Tauripampa, Baños del Inca (locality B), Yanamarca and Chaquicocha, preliminarily classified as *L. viatrix* or *Lymnaea* sp., proved to be *G. truncatula* by ribosomal and mitochondrial DNA markers (Table 1).

##### rDNA ITS-2

All the specimens showed identical ITS-2 sequence, of 401 bp and a 59.10% GC content. When compared with the three ITS-2 haplotypes of *G. truncatula* available in EMBL (H1, H2, H3), this sequence proved to be identical to the previously described ITS-2 haplotype 1 (H1) for *G. truncatula* [EMBL: AJ243017].

##### rDNA ITS-1

Similarly, all specimens studied showed identical ITS-1 sequence, of 504 bp and a 57.74% GC content. This sequence was compared with the four ITS-1 haplotypes of *G. truncatula* available in EMBL (HA, HB, HC) and GenBank (HD) and proved to be different. It has consequently been ascribed to the new haplotype G.tru HE [EMBL: HE610430].

**Figure 2:**
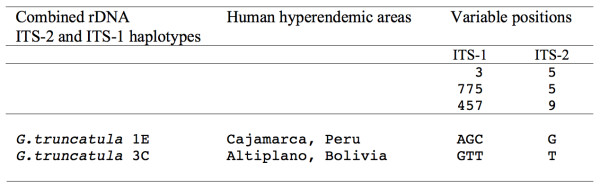
**Comparison of the combined rDNA ITS-2 and ITS-1 haplotypes of *Galba truncatula* of the human fascioliasis hyperendemic areas of Cajamarca and the Bolivian Altiplano.** Nucleotide differences between the combined haplotype 1E representing the "valley transmission pattern" in Cajamarca and the combined haplotype 3C representing the "altiplanic transmission pattern" in the Northern Bolivian Altiplano. Position = numbers (to be read in vertical) refer to variable positions obtained in the concatenated ITS-1 and ITS-2 alignment made with MEGA 5.0

A comparison between the combined rDNA ITS-2 and ITS-1 haplotypes of *G. truncatula* present in Cajamarca, Peru (G.tru CH1E) and the Northern Bolivian Altiplano (G.tru CH3C) shows interesting information about the nucleotide differences shown by this lymnaeid vector in the two human fascioliasis endemic areas with the highest prevalences known (Figure 2).

##### mtDNA 16S rRNA gene

Only one halotype was detected in the specimens from the six different populations studied. This partial sequence was 425 bp-long, presented a biased AT content of 68.70%, and was noted as G.tru-16S-B provisional haplotype [EMBL: HE610432]. Differences with haplotype 16S-A, present in Europe and in the Northern Bolivian Altilplano endemic area, are restricted to only one mutation A/T in position 345 of 16S-A/16S-B haplotype respective alignment.

##### mtDNA cox*1*

**Figure 3:**
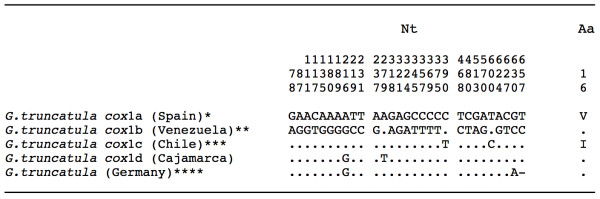
**Differences found in the mtDNA *cox* 1 gene sequence of the *Galba truncatula* populations from Cajamarca and other countries.** Position = numbers (to be read in vertical) refer to variable positions obtained in the alignment made with MEGA 5.0. Nucleotides = Nt; amino acids = Aa; Identical = .; Indel = −. Haplotype codes only provisional due to incomplete sequences of the gene: * [EMBL: AM494011]; ** [GenBank: JF461487]; *** [GenBank: JN051372]. **** sequence shorter than the partial sequence here obtained: no haplotype ascribed [GenBank: EU818799]

Only one haplotype was detected, being identical in all specimens analysed and including 672 bp and a 68.45% of AT content. This sequence was compared with the *cox*1 haplotypes of *G truncatula* known so far and proved to be different. Hence, it is here noted as the new provisional haplotype G.tru-*cox*1d [EMBL: HE610435]. Nucleotide and amino acid differences between the five described haplotypes for *G. truncatula* are listed in Figure 3.

#### Lymnaea neotropica

Specimens from the lymnaeid population collected in the locality of Valle de Condebamaba, previously identified as *L. viatrix*, proved to be *L. neotropica* by ribosomal and mitochondrial DNA markers (Table 1).

##### rDNA ITS-2

All the specimens sequenced showed identical ITS-2 sequence, of 415 bp and a 56.87% GC content. When compared with the ITS-2 haplotypes of *L. neotropica* available in GenBank (H1 and H2), the Peruvian sequence proved to be identical to the previously described ITS-2 haplotype 1 (H1) for *L. neotropica* of its type locality [EMBL: AM412225] (Table 1).

##### rDNA ITS-1

All specimens presented the same ITS-1 sequence of 533 bp and a 56.66 GC content. This sequence was compared with the ITS-1 haplotype of *L. neotropica* available in GenBank (L.neo-HA) and proved to be identical. This haplotype was previously reported in the type locality of Rio Lurin, Lima, Peru and also in Argentina [EMBL: AM412228].

##### mtDNA 16S rRNA gene

Only one halotype was detected in the specimens studied from this population. This partial sequence was 425 bp-long, presented a biased AT content of 69.48%, and was described as L.neo-16S-A provisional haplotype, as it proved to be identical to the one found in the type locality of this lymnaeid species [EMBL: HE610433].

##### mtDNA cox*1*

**Figure 4:**
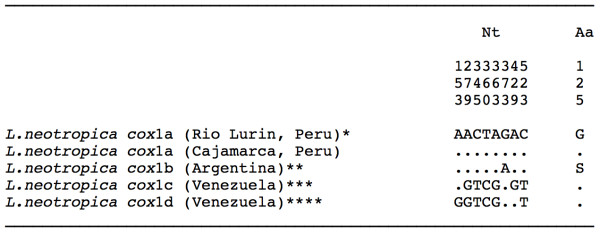
**Differences found in the mtDNA *cox* 1 sequence of *Lymnaea neotropica* from Cajamarca and other countries.** Position = numbers (to be read in vertical) refer to variable positions obtained in the alignment made with MEGA 5.0. Nucleotides = Nt; amino acids = Aa; Identical = .; Indel = −. Haplotype codes only provisional due to incomplete sequences of the gene. * [EMBL: AM494008]; ** [GenBank: FN356741]; *** [GenBank: JF461485]; **** [GenBank: JF461486]

All of the specimens sequenced showed identical *cox*1 nucleotide sequence, of 672 bp and with a biased AT content of 69.5%. This sequence was compared with the four *cox*1 haplotypes of *L. neotropica* known so far and proved to be identical to the previously described haplotype L.neo-*cox*1a from the type locality of this species [EMBL: AM494008]. Nucleotide and amino acid differences between the four described haplotypes for *L. neotropica* are listed in Figure 4.

#### Lymnaea schirazensis

Snail specimens collected from the locality of Baños del Inca (locality A), preliminarily identified as *L. viatrix,* proved to be *L. schirazensis* after ribosomal and mitochondrial DNA marker sequencing (Table 1).

##### rDNA ITS-2

All the specimens presented the same ITS-2 sequence, of 436 bp and a 53.90% GC content. When compared to the two ITS-2 haplotypes of *L. schirazensis* available in GenBank, it proved to be identical to the previously described L.schi-H1 [GenBank: JF272601].

##### rDNA ITS-1

Similarly, all the lymnaeid individuals showed identical ITS-1 sequences, of 533 bp long and a 59.91% GC content. This haplotype was compared with two ITS-1 haplotypes of *L. schirazensis* available in GenBank (HA, HB) and proved to be the same as the previously described L.schi-HB [GenBank: JF272604].

##### mtDNA 16S rRNA gene

Only one halotype was detected in the specimens studied from this population. This partial sequence was 425 bp-long, presented a biased AT content of 69.48%, and corresponded to the provisional haplotype L.schi-16S-A [GenBank: JF272605].

**Figure 5:**
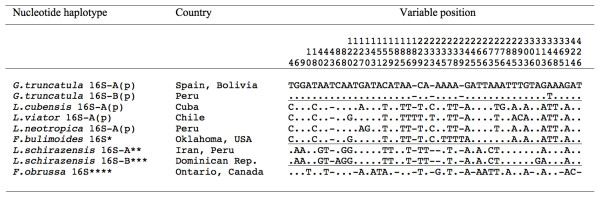
**Nucleotide differences found in the mtDNA 16S gene sequence of *Galba/Fossaria* species and populations studied from Cajamarca and other countries.** Position = numbers (to be read in vertical) refer to the 50 variable positions obtained in the 426-bp-long alignment made with MEGA 5.0. Identical = .; Indel = −. Haplotype codes only provisional due to incomplete sequences of the gene. (p) = new haplotypes obtained in the present study. * [GenBank: AF485657]; ** [GenBank: JF272605]; *** [GenBank: JF272605]; **** [GenBank: AF485658]. Horizontal lines delimitate groups of proximal or similar sequences

The nucleotide differences between the several haplotypes described for different *Galba/Fossaria* species, including *L. schirazensis* plus *G. truncatula, L. neotropica, L. viator, L. cubensis, F. bulimoides* and *F. obrussa* are listed in Figure 5. The most proximal species are *L. neotropica* and *L. cubensis*, differing in only one polymorphic site in position 130.

##### mtDNA cox*1*

All of the specimens sequenced showed identical *cox*1 nucleotide sequence, of 672 bp and with a biased AT content of 69.1%. This sequence was compared with the four *cox*1 haplotypes of *L. schirazensis* known so far and proved to be identical to the previously described haplotype L.schi-*cox*1d [GenBank: JF272610].

### Genetic comparison of lymnaeid species found

Four DNA sequence alignments, one for each marker used, where made to show the total nucleotide differences between the respective haplotypes of the three lymnaeid species present in the fascioliasis hyperendemic area of Cajamarca.

**Figure 6:**
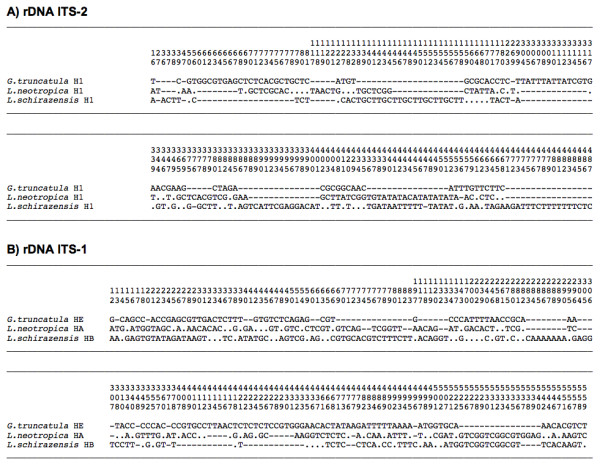
**Nucleotide differences found in the sequences of the rDNA ITS-2 (A) and ITS-1 (B) of the three *Galba/Fossaria* species present in Cajamarca.** Position = numbers (to be read in vertical) refer to variable positions obtained in the alignment made with MEGA 5.0. Alignment manually adjusted. Identical = .; Indel = −

When comparing the ITS-2 sequences, a 487-bp-long alignment was obtained, in which a total of 172 variable positions (35.3%) including mutations and indels appeared (Figure 6A). In the analysis of the ITS-1 sequences, the alignment obtained had a length of 562 bp, with a total of 187 variable positions (33.2%) including mutations and indels (Figure 6B).

**Figure 7:**
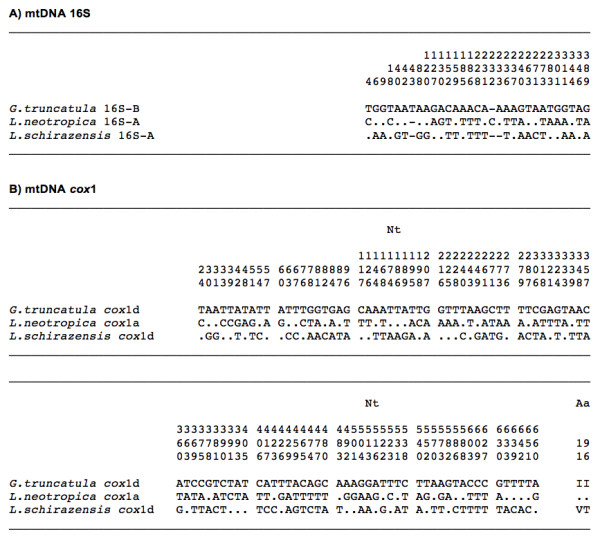
**Differences found in the sequences of the mtDNA 16S ribosomal gene (A) and mtDNA *cox* 1 gene (B) of the three *Galba/Fossaria* species present in Cajamarca.** Position = numbers (to be read in vertical) refer to variable positions obtained in the alignment made with MEGA 5.0. Nucleotides = Nt; amino acids = Aa; Identical = .; Indel = −

In the comparison of the mtDNA 16S sequences, the alignment obtained was 425 bp long, including a total of only 31 variable positions (7.2%), with very few gaps (Figure 7A). In the analysis of the mtDNA *cox*1 sequences, a total of 96 variable positions (14.2%) including only mutations were found in the 672-bp-long alignment. Most of these mutations were however silent, as indeed only two variable positions (0.8%) appeared in the 224-aa-long alignment of the corresponding partial protein sequences (Figure 7B).

### Molecular characterisation of trematode larval stages

The complete sequence of the rDNA ITS-1 obtained from the trematode rediae found in two specimens of *G. truncatula* from Santa Rosa de Chaquil and another two specimens of the same lymnaeid species from Tauripampa is 432 bp long and with a 51.85% GC content. It showed no nucleotide difference when compared with the sequence of that spacer in *F. hepatica* from the Northern Bolivian Altiplano and Spain [EMBL: AJ243016], and thus corresponds to the haplotype code Fh ITS1-HA.

## Discussion

### Lymnaeid species present and their genetic characteristcs

DNA marker sequences prove that there are three different lymnaeid species present in the fascioliasis endemic area of Cajamarca: *G. truncatulaL. neotropica* and *L. schirazensis*. However, previous studies only refered to one lymnaeid species as being responsible for the disease in that area, namely *L. viatrix*[36,37,59-61]. This lymnaeid species appears repeatedly in the Peruvian literature as the main responsible for fascioliasis transmission throughout Peru [62-69]. Indeed, Peruvian *L. viatrix* refer to *L. viator* variety B elongata described long time ago from the surroundings of Lima [70,71] that recently proved, by multiple DNA marker sequencing, to belong to a new species to which the name *L. neotropica* was ascribed [50]. Thus, the existence of true *L. viatrix*, which corresponds to *L. viator* variety A ventricosa [70,71], has so far only been molecularly verified in Argentina [50] and Chile [21]. Additionally, it should be considered that the correct species name for this species is *Lymnaea viator* D’Orbigny, 1835, because the feminine spelling *viatrix* proposed time ago [72] does not fit articles 31.2.1 and 34.2.1 of the International Code of Zoological Nomenclature, as it has been recently highlighted [21].

Unfortunately, it is at present impossible to ascertain whether the populations from Cajamarca classified as *L. viatrix* were in fact *L. neotropica*. These two species are very similar and may be easily confused when only relying on traditional malacological methods [50]. Moreover, both species belong to the Galba/Fossaria group, which is well known due to the pronounced morphological similarity of the numerous species it includes and which makes specimen classification and species differentiation extremely difficult [42]. Consequently, reports on *L. viatrix* in the Cajamarca area may be result of confusion, not only with *L. neotropica*, but also with *G. truncatula* and *L. schirazensis*.

The pronouncedly less numerous nucleotide differences found in the two mtDNA markers 16S and *cox*1 between the three lymnaeid species present in Cajamarca (Figure 7A,B) when compared to their two rDNA markers ITS-2 and ITS-1 (Figure 6A,B) should be highlighted. This result, together with the high number of silent mutations in *cox*1 (Figure 7B), suggests a saturation in both mtDNA markers, a phenomenon already described in lymnaeids [42] and well known in several invertebrate groups [49].

Despite the numerous nucleotide differences in their DNA sequences in both rDNA and mtDNA (Figure 6 and Figure 7), the three lymnaeid species found in Cajamarca may easily be confused one another both in the field and also in the laboratory. Nevertheless, there are several details that may help for at least a preliminary classification (Table 2). However, although a morphological shell trend may help in distinguishing a given species [73], confirmation of the species to which a population belongs can so far only be obtained by DNA sequencing. Moreover, it should be taken into account that mixed populations of *G. truncatula* and *L. schirazensis* have already been described in the field [42]. Hence, care should even be taken when working experimentally in the laboratory.

**Table 2:** Phenotypic characteristics facilitating the classification and differentiation of the lymnaeid snails species present in the fascioliasis hyperendemic area in Cajamarca

**Characteristics**	** *G. truncatula* **	** *L. neotropica* **	** *L. schirazensis* **
Shell:			
- maximum length	12.00 mm	10.36 mm	8.06 mm
- whorls	stepped	convex	regularly convex
- columella	folded	slightly curved and unfolded	straight
Living specimens:			
- Tentacles	wider and with a wide base	*	elongate, slender and with a narrow base
- Eyes	small	*	big and larger
- Colour	mantle roof shows larger unpigmented whitish spots giving a pale appearance to the shell of living specimens by transparency	*	mantle roof from dark brown to blackish throughout, with unpigmented white-greyish round spots, giving a dark appearance to the shell by transparency
Anatomy:			
- Praeputium/penis sheath length ratio	2.50-5.90 mm (mean 3.44 mm)	1.10-3.90 mm (mean 2.12-2.70 mm)	1.20-2.23 mm (mean 1.60 mm)
- Radula	first bilateral teeth tricuspid	first bilateral teeth bicuspid but occasionally tricuspid or rarely quadricuspid	first bilateral teeth mostly bicuspid
Egg clusters:			
- Cluster shape	rounded to oval shape even when containing more eggs	rounded to oval when containing few eggs and lengthening with slightly curved trend when including more eggs	kidney- to banana-like, the more curved, elongated and narrow the more numerous are the eggs inside
- Egg number/cluster	usually 2-15	around 4-16	around 6-14
Ecology:			
- amphibious/terrestr	++	++	+++ (terrestrial trend)
- anthropophyly	+++	+	++
Transmission Capacity:			
- to humans	+++	+	-
- to animals	+++	+++	-

Data from Bargues et al. [42,50], and Khoubbane et al. (unpublished data). Intraspecific variability in lymnaeids of the *Galba*/*Fossaria* group, to which the three species found in Cajamarca belong, is known to be very wide and, thus, characteristics noted in this table should be considered only orientiative. * = comparison in living specimens never performed.

The apparent monomorphic genetic characteristics of each one of the three lymnaeid species in Cajamarca should be highlighted. Each lymnaeid species appear to present only one haplotype for each molecular marker sequenced, although of course additional studies on more populations of these species throughout the endemic area in question are needed to verify this assumption. Anyway, the lack or pronouncedly reduced genetic variability of populations living at very high altitude is known in different organisms and has already been also observed in lymnaeids such as *G. truncatula* in the Northern Bolivian Altiplano [51,74], an area located between 3800 and 4100 m high altitude [2]. This lack of genetic variability of *G. truncatula* in the Northern Bolivian Altiplano is related to the usual selfing trend followed by this lymnaeid [75]. Autofecundation has been verified to be the normal fecundation process in *L. schirazensis* and has also been observed in other species of the *Galba*/*Fossaria* group [42].

The aforementioned monomorphic genetic characteristics suggest an introduction of each one of the three lymnaeid species from only one source. In the case of *G. truncatula*, although its European origin appears evident [1,51], the unexpected new haplotypes of ITS-1 (G.tru-HE), 16S (G.tru-16S-B) and *cox*1 (G.tru-*cox*1d) found in this species in Cajamarca pose a problem when attempting to elucidate the direct geographical source. In South America, the presence of *G. truncatula* has already been molecularly verified in the Northern Altiplano of both Bolivia [51] and Peru [10], Argentina [76,77], Chile [21] and Venezuela [22]. In Colombia, the only report of *G. truncatula* published so far [78] appears to be a misclassification [42].

*Lymnaea neotropica*, originally described from Lima and surroundings [50], appears to be a species restricted to South America but with a very broad geographical distribution covering from Argentina in the Southern Cone [52] up to Venezuela [22]. The combined rDNA and mtDNA haplotype of this species in Cajamarca is identical to the one from the type locality. The presence of this combined haplotype in both Peruvian areas may probably be related to the livestock trade between Lima and Cajamarca which was very intense along the old rural Inca routes at a given period of the early Spanish colonisation [79].

The combined rDNA and mtDNA haplotype of *L. schirazensis* present in Cajamarca appears to be a mix. It shares the ITS-2 haplotype L.schi-H1 with Spain, the Dominican Republic and Venezuela, and is different from the one in Rio Lurin, close to Lima. Its ITS-1 haplotype L. schir-HB has also been found in Mexico and Ecuador and in Peru it is present in both Cajamarca and Rio Lurin. The ribosomal 16S gene of the mtDNA does unfortunately not furnish any biogeographical information, as the haplotype L.schir-16S-HA found in Cajamarca is the same as everywhere. And finally, the mtDNA *cox*1 haplotype L.schir-*cox*1d appears to be unique [42]. Although a trans-Andean livestock introduction route was launched between western Andean Venezuela and the Colombian Bogota and also further southward [80], the introduction of *L. schirazensis* with livestock and humans should have most probably occurred from northern Peru, probably by livestock transported between Quito and Lima through Cajamarca along the old rural Inca routes followed by the Spanish conquerors [42].

### Implications for fascioliasis transmission and epidemiology

Fascioliasis in Cajamarca has been the focus of several studies on both livestock [36,37,61,81] and humans [12,13,82-84]. According to results from these studies, Cajamarca is a typical representative of the "valley transmission pattern" of fascioliasis [1,7,81] and the area presenting the highest prevalences in children, both in average (24.4%) and local maximum (47.7%), among all human endemic areas in Peru [13]. With regard to human prevalences, Cajamarca is only surpassed by the Northern Bolivian Altiplano [2,19]. Interestingly, only one lymnaeid species, *G. truncatula*, is responsible for such a human hyperendemic situation in the Northern Altiplano [51], whereas DNA sequence results here obtained demonstrate that there are three different lymnaeid species present in the hyperendemic area of Cajamarca.

**Figure 8:**
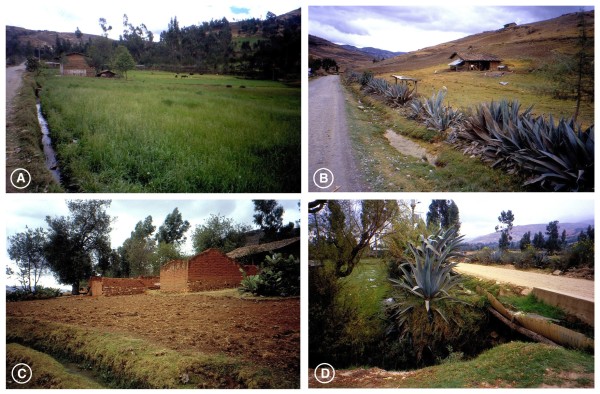
**Biotopes of *Galba truncatula* in Cajamarca.** Environments of localities where *Galba truncatula* populations were collected: **A**) Encañada, Encañada district; **B**) Santa Rosa de Chaquil, Encañada district; **C**) Tauripampa, Llacanora district; **D**) Yanamarca, Jesus district

Among the three lymnaeid species present in Cajamarca, *Galba truncatula* is considered the best *F. hepatica* transmitter known, with such a parasite/vector interaction as to support that this lymnaeid may be considered the original vector of this fasciolid [1]. Moreover, *G. truncatula* is known to give rise to very high human infection rates, both prevalences and intensities, at the very high altitude of the Northern Altiplano of both Bolivia [2,18,19] and Peru (10). Such high fascioliasis transmission rates have been proved to be the consequence of life cycle modifications in both *F. hepatica* and *G. truncatula* as an adaptation response to the extreme conditions of the very high altitude [51]. Several aspects suggest that *G. truncatula* may behave in the 2600–3100 m high altitudes of Cajamarca similarly to in the 3800–4100 m altitudes of the Northern Altiplano, namely:

 a) the results of the field surveys carried out showing that populations of this species are the most abundant in the endemic area;

 b) the typical anthropophylic characteristics of this lymnaeid appeared evident when proving to be the usual snail species in the neighbourhood of human communities such as Encañada, Santa Rosa de Chaquil, Tauripampa and Yanamarca (Figure 8), close to localities where schoolchildren appeared infected [13];

 c) the high population densities of thois species found in the high altitude localities studied (Table 1);

 d) the finding of infected specimens in two populations of this lymnaeid by means of the DNA sequencing methods (Table 1), despite the relatively few specimens analysed for infection detection (prevalences in lymnaeids are usually low).

In front of the aforementioned similarities, the genetic differences between *G. truncatula* populations from two different environments such as Cajamarca and the Northern Bolivian Altiplano should be emphasized (Figure 2). Whereas in Cajamarca the disease transmission follows a seasonality mainly related to the cyclic temperatures [37], in the Northern Altiplano fascioliasis transmission takes place throughout the year due to (i) the scarcely varying temperatures [8] and (ii) the link of *G. truncatula* populations to permanent water bodies as the consequence of the absence of sufficiently long-standing temporal water bodies due to the high evapotranspiration rates [2,8].

*Lymnaea neotropica* has also been found to be linked to human infection. This species was originally described from the surroundings and other areas near Lima, Peru [50], where human infection has repeatedly been detected [14,85]. Although none of the *L. neotropica* specimens sequenced in Cajamarca was infected by *F. hepatica*, Peruvian *L. viatrix* (= *L. viator*) variety B elongata (= *L. neotropica* according to [50]) has been shown to transmit fascioliasis both experimentally and in nature [62,67,86]. The transmission capacity of this lymnaeid vector has also been molecularly confirmed in Argentina, where it appeared linked to animal infection [52], as in Venezuela [22].

With regard to *L. schirazensis*, experimental infection assays of different geographical strains of this snail species from different continents with *F. hepatica* and *F. gigantica* have proved that fasciolid larval stages are not able to fully develop within this lymnaeid, which does therefore not participate in disease transmission [42]. A large multidisciplinar study has demonstrated that *L. schirazensis* has an outstandingly broad distribution from lowlands to highlands, including from below sea level (−23 m) in Iran up to very high altitude areas in Ecuador (3158 m). Throughout this wide altitudinal range, it has always been confused with *G. truncatula* in the Old World and with *G. truncatula* and other similar *Galba*/*Fossaria* vector species in the Americas. This hitherto overlooked species has been masking not only the geographical distribution of fascioliasis, but also fasciolid population specificity/susceptibility analyses. It shall be considered that *L. schirazensis* has been shown molecularly to be able to cohabit with other *Galba*/*Fossaria* group species in such proximity that a natural population of specimens of an apparently unique lymnaeid species may in fact involve specimens from two different species unnoticeably mixed. Such mixed populations have already been described in the case of *G. truncatula* and *L. schirazensis*[42].

## Conclusions

Sequences of the rDNA and mtDNA markers indicate that two lymnaeid vectors, *G. truncatula* and *L. neotropica*, and a third non-transmitting lymnaeid, *L. schirazensis*, inhabit the fascioliasis hyperendemic area of Cajamarca. This new scenario pronouncedly changes the situation described so far, in which disease transmission was mentioned to be related to only one lymnaeid species, *L. viator* (= *L. viatrix*). All suggests that this has been the consequence of misclassifications due to the inaccuracy of traditional malacological methods for species differentiation among the *Galba*/*Fossaria* group of lymnaeids to which the three aforementioned, morphologically similar, small snail species belong [42,50].

The nearby detection of populations of *G. truncatula* and *L. schirazensis* in Baños del Inca district, and of *G. truncatula* and *L. neotropica* in Cajabamba district, indicate that these three lymnaeid species geographically overlap inside the hyperendemic area. This fact poses two problems of increasing difficulty for epidemiological studies and control action.

First, there is a problem in classifying lymnaeid specimens in both field and laboratory activities in Cajamarca. Indeed, although several phenotypic characteristics may a priori be helpful for a preliminary specimen classification (Table 2), a definitive classification of a specimen can only be obtained by the sequencing of at least one of the molecular markers here used, ITS-2, ITS-1, 16S and *cox*1. This problem becomes extremely important given the transmission capacity differences of these three lymnaeid species: *G. truncatula* as the vector mainly involved in disease transmission to humans, *L neotropica* as a typical responsible for livestock infection, and *L. schirazensis* as a lymnaeid unable to transmit fascioliasis. Moreover, from the ecological point of view, these three species may be found to show similar amphibious characteristics. Additionally, *L. schirazensis* pronouncedly increases the confusion problem, owing to its ability to mix its specimens and populations with those of other *Galba*/*Fossaria* species and distort fascioliasis data such as transmission capacity and infection susceptibility. Such *L. schirazensis* mixing has already been described with *G. truncatula*[42].

Second, the complexity of the overlap of two different lymnaeid vector species poses a serious problem for the development of methods which have shown to be useful for epidemiological analysis, surveillance and control of human fascioliasis in Andean high altitude endemic areas, such as mathematical modelling through the application of different climatic indices (Mt, Wb-bs) [8], and Remote Sensing (RS) with Geographical Information Systems (GIS) [9]. Thus, both methods appear to be useful to monitor the fascioliasis situation in the Northern Bolivian Altiplano, where only one lymnaeid species, *G. truncatula*, is responsible for disease transmission. However, the same methods did not appear to be sufficiently accurate when analysing fascioliasis in the central regions of Chile where human fascioliasis is endemic and livestock prevalences are the highest in the country [87]. Recent DNA sequencing results have shown that the endemic regions of Chile where the RS-GIS method did not appropriately work were in fact areas where there is an overlap of two different lymnaeid vector species with supposedly different transmission capacity [21]. The same problem may be expected in Cajamarca if similar low resolution mapping is applied.

These conclusions should be considered within future control activities in the province of Cajamarca. In this hyperendemic area, a pilot intervention to assess human treatment strategies was successfully performed in 2007 and 2008, human fascioliasis treatment activities have since then been yearly implemented, thanks to the availability of Egaten® (triclabendazole for human use; donation by Novartis Pharma AG), provided by WHO through the Ministry of Health in Lima and the Dirección Regional de Salud of Cajamarca, and a large initiative for animal control to diminish risk infection has already been approved.

## Competing interests

The authors declare that they have no competing interests.

## Authors’ contributions

MDB contributed to the design of the study, participated in field collections, analysed the sequences, and helped to draft the manuscript. PA carried out the DNA sequencing processes. MK performed the phenotypic characterisation. PO participated in laboratory procedures and facilitated logistics for both experimental and field work in the endemic areas of Cajamarca. CN participated in lymnaeid collections in the endemic areas of Cajabamba. SMC designed and supervised the study, participated in field collections, performed the epidemiological analyses, and wrote the manuscript. All authors read and approved the final manuscript.
